# Effects of different warm-up methods on 50-meter breaststroke swimming performance

**DOI:** 10.3389/fbioe.2024.1505648

**Published:** 2025-01-07

**Authors:** Hsuan-Yen Lee, Te Chao, Chi-Chieh Hsu, Ning-Wei Chang, Yi-Liang Chen, Yung-Shen Tsai

**Affiliations:** ^1^ Graduate Institute of Sports Training, University of Taipei, Taipei, Taiwan; ^2^ Graduate Institute of Sports Science, University of Taipei, Taipei, Taiwan; ^3^ Department of Aquatic Sports, University of Taipei, Taipei, Taiwan; ^4^ Graduate Institute of Sports Equipment Technology, University of Taipei, Taipei, Taiwan

**Keywords:** dynamic warm-ups, breaststroke specialists, individual medley swimmers, motion analysis, IMU

## Abstract

**Purpose:**

To examine the effects of different warm-up methods on 50 m breaststroke performance in both breaststroke specialists and individual medley swimmers.

**Methods:**

18 swimmers (breaststroke group: 9, individual medley group: 9) who met the qualification standards for the National Intercollegiate Athletic Games participated in this study. Each participant completed four different warm-up protocols (a conventional 1,400 m warm-up and a 700 m conventional warm-up that integrated tubing-assisted (TA), paddle (PD), or squat (SQ) warm-ups) over four separate days. Following each warm-up protocol, a 50 m breaststroke performance test was conducted with inertial measurement unit (IMU) sensors attached to specific body segments to evaluate and compare stroke performance, stroke length, stroke frequency, and the acceleration of the hands, sacrum, and feet across different warm-up methods.

**Results:**

The breaststroke specialists who performed the TA warm-ups recorded significantly less time than those who performed the conventional 1,400 m warm-ups (35.31 ± 1.66 s vs. 35.67 ± 1.83 s, *p* = 0.006). There was a trend that individual medley specialists who performed the SQ warm-ups recorded less time than those who performed the PD warm-ups (34.52 ± 1.45 s vs. 34.92 ± 1.46 s, *p* = 0.043). The stroke length of breaststroke specialists following the TA warm-ups was shorter than that following the PD warm-ups, the SQ warm-ups, and the conventional 1,400 m warm-ups. Breaststroke specialists who engaged in the TA warm-ups had higher stroke frequency than those who engaged in the conventional 1,400 m warm-ups, the SQ warm-ups, and the PD warm-ups. During the TA warm-ups, breaststroke specialists exhibited a shorter stroke length and a higher stroke frequency than individual medley specialists. Acceleration data from the center of mass and limb segments, recorded by IMUs, were insufficient to fully explain the variations in stroke frequency, stroke length, and overall performance caused by the different warm-up protocols.

**Conclusion:**

Breaststroke specialists exhibited significant improvement in their 50 m breaststroke performance after the TA warm-up. By contrast, individual medley specialists benefited more from the SQ warm-up.

## 1 Introduction

In international short-distance swimming competitions, a difference of one-hundredth of a second often determines the outcome of a race. For example, during the 2022 World Aquatics Championships, the difference between the first and second place in the men’s 50-m breaststroke was only 0.03 s (www.fina.org). In competitions at this level, any change that results in slight variations in speed must be seriously considered (Stewart and Hopkins, 2000). Such variations in speed are vital to breaststroke swimmers, which Kolmogorov et al. (1997) indicated has the poorest hydrodynamics and the highest resistance of all swimming styles. Therefore, effectively improving metrics such as range of motion, speed, and power in breaststroke is crucial for enhancing competitive performance.

Athletes commonly use dynamic warm-ups to enhance performance. Such warm-ups can be adjusted by varying the resistance of exercises to achieve varied intensities and neuromuscular adaptations (Bishop, 2003; Neiva et al., 2014). Unlike static stretching, dynamic warm-ups can increase the range of motion without negatively affecting maximum strength and explosiveness (Behm et al., 2016). The improvement in performance from dynamic warm-ups is due to increased muscle temperature, increased muscle blood flow, and enhanced motor unit recruitment (Bishop, 2003; Neiva et al., 2014).

In practice, swimmers often use hand paddles for sprint warm-ups, similar to weighted warm-ups on land that provide a stimulus to enhance performance during competition. Most coaches believe this can improve swimmers’ stroke length and speed. These methods are like those employed by athletes in other sports in that they involve increasing resistance in movements that mimic competition actions, such as performing weighted lunges, engaging in sled sprints, and adding weighted bats (Beato et al., 2020; Reyes and Dolny, 2009; Seitz et al., 2017). Furthermore, this concept is not limited to using movements similar to those performed during competition. Incorporating resistance training exercises into the warm-up can compel motor units to recruit more muscle fibers (Conrado de Freitas et al., 2021), thereby enhancing the athlete’s explosive performance (Brown et al., 2023; Esformes and Bampouras, 2013). For example, Esformes and Bampouras (2013) observed that performing squats at 3 repetition maximum (RM) intensity improved countermovement jump (CMJ) performance after 5 min. In swimming competitions, more and more coaches regard this warm-up method as a way to enhance stroke distance and incorporate it into pre-race preparations. Other methods involve reducing resistance during dynamic warm-ups, commonly seen in swimming as well, where resistance tubes are used to propel athletes in the direction of a sprint. This technique aims to help swimmers achieve faster movement speeds or higher stroke frequencies during warm-ups, with the intention of translating this advantage into their performance in swimming competitions. This strategy is similar to assisted jumps in land-based sports, allowing athletes to move with less resistance and increasing their movement speed (Sheppard et al., 2011). Assisted training works by reducing the athlete’s body weight, thereby overcoming inertia during the movement process to achieve faster speeds during the power output phase, known as overspeed (Sheppard et al., 2011; Stien et al., 2020). This effect enables athletes to recruit muscles more rapidly during competition (Cazas et al., 2013). In addition to the traditional long-distance swimming warm-up methods, coaches are using the three aforementioned methods to warm up their athletes. However, there has yet to be a clear comparison of how these warm-up methods affect swimmers’ performance in the 50-m breaststroke event.

Various instruments have been utilized to analyze swimming movements. Three-dimensional (3D) motion analysis captures movement in three-dimensional space. It provides a comprehensive understanding of motion, including rotations and depth. Two-dimensional (2D) motion analysis tracks motion in a two-dimensional plane. It is commonly used for planar movements where depth is not included. Underwater 3D analysis systems are considered the most reliable and valid by numerous scholars and coaches (Chung and Ng, 2012). However, due to the cost and limited availability of underwater 3D analysis systems, most coaches currently use 2D methods for underwater movement analysis (Cortesi et al., 2019). 2D analysis requires synchronizing a sagittal plane camera and a frontal plane camera and manual identification of joint positions. This complex method poses challenges to accurate and efficient recording (Ceseracciu et al., 2011). In recent years, a simpler method based on inertial measurement units (IMUs) has been employed for swimming movement analysis. IMUs can be used to continually analyze and monitor the swimming process and are not confined to a single space, substantially enhancing their applicability (Dadashi et al., 2012; Mooney et al., 2015).

Several studies have used IMUs to interpret variations in center of mass and segmental instantaneous velocities during freestyle swimming, verifying their accuracy in detecting intracycle variability (Cortesi et al., 2019; Dadashi et al., 2012). However, few experiments have utilized IMUs to analyze breaststroke movements. Gonjo et al. (2022) employed video analysis to identify kinematic differences between breaststroke specialists and individual medley swimmers, discovering that breaststroke specialists exhibited greater swimming speed and stroke length, whereas individual medley swimmers exhibited higher stroke frequency, although this difference was nonsignificant. IMU analysis provides athletes with immediate feedback and greatly reduces the spatiotemporal constraints associated with fixed-position cameras.

Any method that can enhance breaststroke performance deserves attention. Although many coaches currently attempt various warm-up methods (such as using hand paddles, deep squats, or tubing-assisted exercises) to improve the performance of breaststroke and individual medley swimmers in competitions, there is a lack of research investigating the effects of these warm-up methods on breaststroke movements and performance. Therefore, the aim of the present study was to compare the effects of various warm-up methods (a conventional 1,400 m warm-up and a 700 m conventional warm-up that integrated tubing-assisted, paddle, or squat warm-ups) on the 50 m breaststroke performance of both breaststroke specialists and individual medley swimmers. The present study also examined the performance enhancements achieved through these warm-up methods for the two types of swimmers and utilized IMU-based kinematic analysis to assess the applicability of IMUs in analyzing breaststroke movement. The authors hypothesized that the use of swim paddles, resistance tubes, and squats would improve 50 m breaststroke kinematic performance. Additionally, the warm-up methods varied in their effects on the movement, strategies, and specializations of breaststroke specialists and individual medley swimmers.

## 2 Materials and methods

### 2.1 Participants

This study recruited 18 swimmers who met the swimming competition standards of the National Intercollegiate Athletic Games. Participants were required to have more than 4 years of swimming training and more than 1 year of professional strength training. This ensured that they could quickly familiarize themselves with our tests without requiring a long adaptation period. Additionally, participants were excluded if they had major musculoskeletal injuries, cardiovascular diseases, diabetes, or other medical conditions or surgeries within the 6 months prior to this study. Furthermore, participants were required to refrain from consuming alcohol or caffeine-containing beverages 24 h prior to each test or training intervention. The anthropometric characteristics of the participants are presented in Table 1.

**TABLE 1:** Anthropometric characteristics of swimmers.

	Breaststroke	Individual medley	Independent
	(n = 9)	(n = 9)	t-test
	mean ± SD	mean ± SD	*p*-value
Age (years)	21.2 ± 2.8	19.6 ± 3.6	0.311
Body height (cm)	173.2 ± 6.5	175.8 ± 10.8	0.534
Body mass (kg)	65.8 ± 5.7	69.7 ± 12.2	0.426
Training experience (years)	11.3 ± 5.0	11.5 ± 4.2	0.929

Prior to the experiment, participants were briefed regarding risks and asked to complete the American College of Sports Medicine’s Exercise Preparticipation Health Screening (Riebe et al., 2015). After completing the assessment, participants signed an informed consent form approved by the Institution Review Board of the University of Taipei. If participants experienced discomfort during the research process, they could withdraw from the study immediately.

### 2.2 Study design

This is a convenience sampling study that focuses on elite athletes. Participants were divided into two groups based on their specialization: a breaststroke group (n = 9; male = 7, female = 2) and an individual medley group (n = 9; male = 9). Each participant completed four types of warm-up protocols, including a conventional 1,400 m warm-up and a 700 m conventional warm-up that incorporated tubing-assisted (TA), paddle (PD), or squat (SQ) warm-ups. Following each warm-up protocol, a 50 m breaststroke performance test was conducted to evaluate and compare stroke performance, stroke length, stroke frequency, and the acceleration of the hands, sacrum, and feet across the different warm-up methods.

### 2.3 Procedures

Over the course of five visits to the research facility, with each visit spaced 1 week apart, participants completed a 1-repetition maximum (1 RM) strength test, followed by four swimming warm-up protocol trials. Initially, participants performed a 1,400 m warm-up swimming test. Subsequently, they completed the remaining warm-up swimming tests according to a counterbalanced design: the first participant in each group followed the order TA, PD, and SQ; the second participant completed the tests in the order PD, SQ, and TA; and the third participant used the sequence SQ, TA, and PD. This systematic rotation ensured that all participants underwent each warm-up protocol in a balanced and unbiased manner.

### 2.4 1 RM strength test

The 1 RM strength test was conducted according to the barbell back squat strength testing standards of the American College of Sports Medicine. Initially, participants performed two sets of between 8 and 10 warm-up repetitions at between 50% and 80% of their self-determined maximum intensity. Following the warm-up, the 1 RM test began with a weight that was 5% less than the participant’s perceived maximum strength. After each successful squat, the weight was increased by a maximum of 2%. Participants rested for 3 min between each attempt, and the maximum strength value was determined on the basis of between 3 and 6 attempts (Thompson et al., 2013).

### 2.5 Warm-up interventions

**FIGURE 1:**
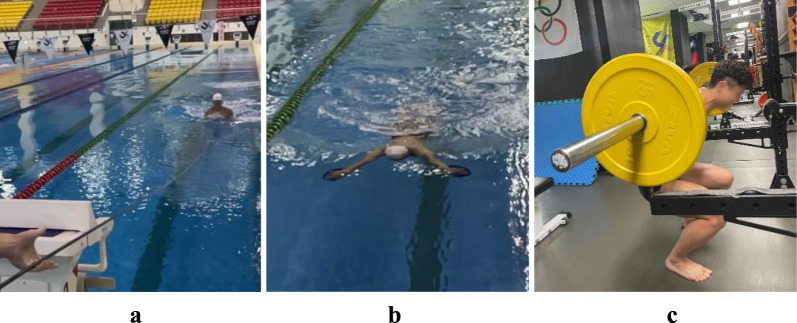
Different warm-up methods. **(A)** Tubing assisted (TA) **(B)** Paddle (PD) **(C)** Squat (SQ).

The warm-up protocol for this study was based on that in earlier studies. Each of the four warm-up methods was completed within 25 min. The conventional 1,400 m warm-up included several swimming techniques and paces: 400 m of swimming using a stroke and pace of choice; 200 m of pulling exercises (25 m steady/25 m fast); 200 m of kicking exercises using fins (15 m fast/35 m steady); four sets of 100 m, alternating between two pulling exercises and two individual medleys, with a 10 s rest between each set; 100 m of simple swimming; and two sets of 50 m consisting of diving and swimming with a pace of 15 m fast/35 m easy (Ng et al., 2020). Before engaging in the TA, PD, and SQ warm-ups, participants completed a 700 m standard water warm-up, comprising 200 m of swimming with any stroke at any pace; 100 m of pulling exercises (25 m steady/25 m fast); 100 m of kicking exercises using fins (15 m fast/35 m steady); two sets of 100 m, comprising one pulling exercise and one individual medley, with a 10 s rest between each set; 50 m of relaxed swimming; and 50 m consisting of diving and swimming alternating 15 m fast/35 m relaxed (Cuenca-Fernández et al., 2020; Ng et al., 2020). Following the water warm-up, participants rested for 5 min before performing each of the following three warm-up exercises: (1) TA Warm-up: Two 20 m TA swims, with a 2-min rest between each. During the TA warm-up, one end of a tube (StrechCordz^®^ resistance band, NZ Manufacturing, Tallmadge, OH, USA) was secured to the swimmer’s waist, while the other end was pulled by a researcher at the finish line as quickly as possible during the swimmer’s upper limb pull phase and lower limb kick phase (Figure 1A). (2) PD Warm-up: Two 20 m sprints were performed using swim paddles (Strokemakers Technique Swimming Paddles, Strokemakers, Phoenix, AZ, USA) slightly larger than the palms of the swimmers’ hands for the breaststroke, with a 2-min rest between each sprint (Figure 1B). (3) SQ Warm-up: Two sets of three repetitions of barbell back squat at 85% of 1 RM, with a 2-min rest between sets (Figure 1C).

### 2.6 Swimming test

The 50 m breaststroke swimming test was conducted in an international-standard 50 m swimming pool and timed using a SEIKO swimming timing system and a starting pistol. Two cameras (120 Hz with a resolution of 1080p) (GoPro Hero6, GoPro, Inc., San Mateo, CA, United States) were positioned poolside to record the time from the dive start to the 15 m and 25 m marks. These two time points represent the swimmers’ starting ability and performance during the first half of the race. The flashing light of the starting pistol synchronized all cameras. After the test, Kinovea video analysis software (v. 0.8.26, Kinovea, Paris, France) was used to analyze the split time at 15 m and 25 m. During the breaststroke test, IMUs (Xsens DOT, Movella Inc., Henderson, NV, United States) were employed to record the maximum forward and backward accelerations of the hands, the maximum forward acceleration of the sacrum, and the maximum backward acceleration of the feet at a frequency of 120 Hz. For stroke phase analysis, an iPhone 13 (Apple, United States) was used to record at 120 frames per second (FPS) with a resolution of 1080p in the slow-motion video section, capturing the distance from the dive start to the 50 m mark along the poolside. A custom piezoelectric synchronization device was connected to a nonattached IMU and a light-emitting diode (LED) device. Pressing the piezoelectric synchronization device caused the IMU to shake and the LED device to flash, enabling the mobile phone to capture both actions simultaneously for precise synchronization. The swimming test subsequently commenced to enable the mobile phone to record swimming motions and phases, the IMUs to collect data, and stroke rates and stroke lengths to be calculated.

### 2.7 Data processing

Video analysis was conducted using Kinovea, an open-source software program, to analyze footage from the 15 m and 25 m marks. The starting point for timing was the flash of the starting pistol, and the end point was when the swimmer’s fingertip touched the 15 m or 25 m mark. The split time for the segment between 15 and 25 m was calculated by subtracting the 15 m split from the 25 m split, and the split time for the segment between 25 and 50 m was calculated by subtracting the 25 m split from the 50 m split. Additionally, video recordings of the entire 50 m breaststroke were used to determine stroke rate and stroke length for the segments between 15 and 25 m and between 25 and 50 m. Stroke rate was calculated by dividing the number of stroke cycles completed by the time taken for each segment, whereas stroke length was calculated by dividing the segment distance by the number of stroke cycles completed. A single stroke cycle was defined starting from the arm glide phase, where the arms were fully extended forward until they began moving backward, and concluding with the recovery two arms phase, where the arms returned forward to a 90° angle relative to the forearm and the arms were again fully extended (Strzala et al., 2013).

**FIGURE 2:**
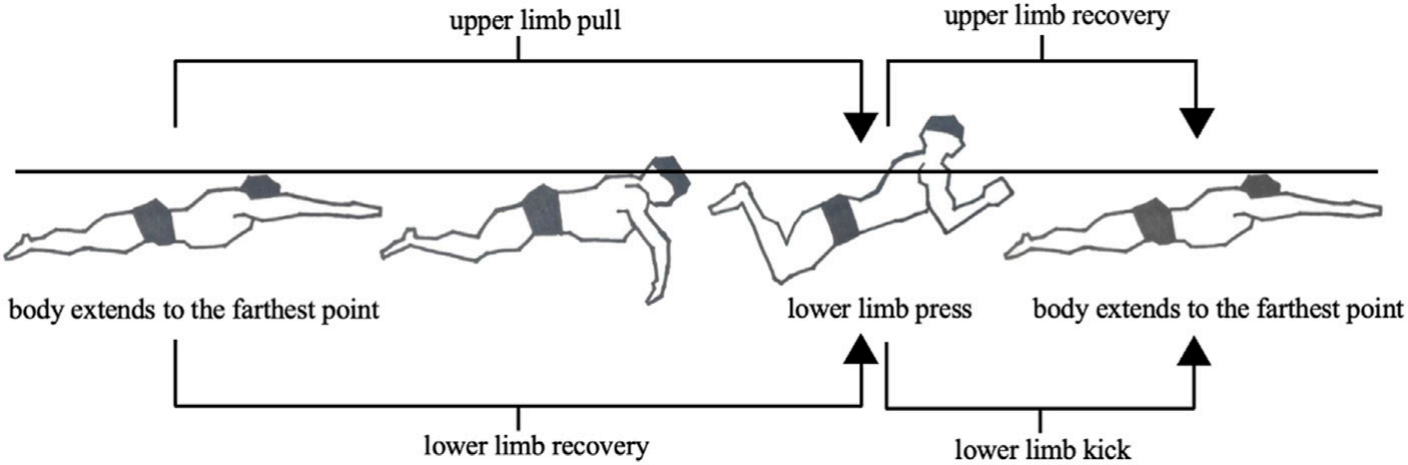
Phases of breaststroke movements.

IMU data were collected to analyze the forward acceleration of the hands, sacrum, and feet over three complete stroke cycles closest to the 15 m, 25 m, and 50 m marks. The accelerations of the hands and feet were standardized relative to the forward acceleration of the sacrum. The average maximum accelerations of the hands, feet, and sacrum during the three stroke cycles at 15, 25, and 50 m were used for statistical analysis. The phases of the breaststroke cycle, illustrated in Figure 2, involve the body extending to the farthest point and transitioning to the upper limb pull and lower limb recovery phase, following which the foot pushes backward as the upper limb recovers and enters the lower limb kick phase before the body extends again to its farthest point.

### 2.8 Statistical analysis

All data were analyzed using SPSS (IBM SPSS Version 25.0, Chicago, IL). G*Power was utilized to calculate the required sample size for a two-group design with four measurements, using an effect size of 0.5, an alpha level of 0.05, and a power of 0.8. The analysis type was “ANOVA: Repeated measures, within-between interaction,” with an assumed moderate correlation among measures (0.5) and a nonsphericity correction factor of 1 (Faul, et al., 2009). Based on these parameters, G*Power determined that each group would need 8 participants. However, due to the availability of participants and to enhance the robustness of the findings, 9 participants were recruited per group. Shapiro-Wilk tests were performed to assess the normality of the data distribution. If the data did not follow a normal distribution, non-parametric analysis was employed. A two-way mixed-design ANOVA (4 warm-ups * 2 groups) was employed to compare the effects of the four warm-ups (1,400 m, TA, PD, SQ) on the performance of breaststroke and individual medley specialists. If an interaction was observed, a one-way repeated measures ANOVA was performed to compare the differences between warm-ups within each group. Additionally, an independent sample t-test was conducted to examine differences between the two specialties for the same warm-up. Statistical significance was set at α = 0.05. Partial eta-squared (η_p_^2^) values were calculated to assess the effect sizes for main effects and interaction effects, categorized as small (0.01 ≤ η_p_^2^ < 0.06), medium (0.06 ≤ η_p_^2^ < 0.14), and large (η_p_^2^ ≥ 0.14) (Cohen, 2013). In the presence of a significant interaction effect, a Bonferroni *post hoc* test was conducted to identify specific differences, accounting for the small sample size.

## 3 Results

The time for the 50 m breaststroke test at the 15, 25, and 50 m marks, in addition to the split time, are presented in Table 2. The results of the analysis indicated an interaction between the warm-up protocols and the specialties in the time for the 50 m breaststroke test (F (3, 48) = 2.342, *p* = 0.038, *η*_*p*_^*2*^ = 0.477). Post hoc comparisons revealed that the breaststroke specialists performing the TA warm-ups reached the 15, 25, and 50 m marks in fewer seconds than those performing the conventional 1,400 m warm-ups (*p* = 0.006). Additionally, swimmers performing the PD warm-ups reached the 15, 25, and 50 m marks more rapidly than did those performing the conventional 1,400 m warm-ups (*p* = 0.023) and the squat warm-up (*p* = 0.035). Individual medley specialists who performed the SQ warm-ups reached the marks more rapidly than did those who performed the PD warm-ups (*p* = 0.043). An interaction was observed in the split time for the segment between 25 and 50 m (F (3, 48) = 2.177, *p* = 0.037, *η*_*p*_^*2*^ = 0.417). Post hoc comparisons revealed that breaststroke specialists who performed the TA warm-ups had shorter split time than did those who performed the conventional 1,400 m warm-ups (*p* = 0.037). Finally, the PD warm-ups were associated with shorter split time than the SQ warm-ups (*p* = 0.029).

**TABLE 2:** Split time for 50 m breaststroke race.

Warm-ups	Breaststroke	Individual medley		Two-way ANOVA with mixed design (*p*-value)		
	(n = 9)	(n = 9)		Intervention	Group	Interaction
	Mean ± SD	Mean ± SD				Post hoc
Time to 15 m (sec)				0.051	0.082	0.748
1400 m	8.01 ± 0.58	7.52 ± 0.64				
Tubing assisted	7.89 ±0.77	7.31 ± 0.45				
Paddle	7.93 ± 0.69	7.45 ± 0.44				
Squat	7.68 ± 0.72	7.27 ± 0.35				
15-25 m segment (sec)				0.587	0.577	0.060
1400 m	7.62 ± 0.45	7.70 ± 0.62				
Tubing assisted	7.99 ± 0.51	7.30 ± 0.67				
Paddle	7.76 ±0.74	7.94 ± 0.70				
Squat	7.6± 0.49	7.65 ± 0.72				
Time to 25 m (sec)				0.858	0.109	0.049*
1400 m	15.64 ± 0.90	15.22 ± 0.74				
Tubing assisted	15.87 ± 1.02	14.61 ± 0.78				TA: IM > BR, *p* = 0.011
Paddle	15.69 ± 1.11	15.38 ± 0.85				
Squat	15.58 ± 0.95	15.03 ± 0.78				
25-50 m segment (sec)				0.221	0.997	0.037*
1400 m	20.03 ± 1.12	19.79 ± 0.97				BR: 1400 m > TA, *p* = 0.037
Tubing assisted	19.44± 0.96	20.17 ± 1.18				BR: AQ > PD, *p* = 0.029
Paddle	19.36 ± 1.27	19.53 ± 1.36				
Squat	20.10 ± 1.32	19.49 ± 0.83				
Time to 50 m (sec)				0.186	0.451	0.038*
1400 m	35.67 ±1.83	35.01 ± 1.41				BR: 1400 m > TA, *p* = 0.006**
Tubing assisted	35.31 ± 1.66	34.79 ± 1.38				BR: 1400 m > PD, *p* = 0.023
Paddle	35.05 ± 2.11	34.92 ± 1.46				BR: SQ > PD, *p* = 0.035
Squat	35.67 ±2.17	34.52 ± 1.45				IM: PD > SQ, *p* = 0.043

BR, Breaststroke; IM, Individual medley; TA, Tubing-assisted; PD, Paddle; SQ, Squat.

*p < 0.05, **p < 0.0083 (Bonferroni adjustment for post hoc comparison).

A significant interaction was observed between the warm-ups and the specialties in stroke length (F (3, 48) = 4.453, *p* = 0.048, *η*_*p*_^*2*^ = 0.316) and frequency (F (3, 48) = 6.680, *p* = 0.005, *η*_*p*_^*2*^ = 0.295) from 25 to 50 m (Table 3). Post hoc comparisons revealed that the stroke length of breaststroke specialists following the TA warm-ups was significantly shorter than that following the PD warm-ups (*p* = 0.001), the SQ warm-ups (*p* = 0.001), and the conventional 1,400 m warm-ups (*p* = 0.012). Additionally, the stroke length during the SQ warm-ups was significantly longer than during the conventional 1,400 m warm-up (*p* = 0.008). Furthermore, breaststroke specialists who engaged in the TA warm-ups had significantly higher stroke frequency than those who engaged in the conventional 1,400 m warm-ups (*p* = 0.005), the SQ warm-ups (*p* = 0.001), and the PD warm-ups (*p* = 0.014). The stroke frequency during the conventional 1,400 m warm-ups (*p* = 0.004) and the PD warm-ups (*p* = 0.015) was also higher than that during the SQ warm-ups. Finally, during the TA warm-ups, breaststroke specialists exhibited a shorter stroke length (*p* = 0.010) and a higher stroke frequency than individual medley specialists (*p* = 0.005).

**TABLE 3:** Split stroke length and frequency for 50 m breaststroke race.

Warm-ups	Breaststroke	Individual medley	Two-way ANOVA with mixed design (*p*-value)		
	(n = 9)	(n = 9)	Intervention	Group	Interaction
	Mean ± SD	Mean ± SD			Post hoc
15–25 m Stroke length (m)			0.149	0.075	0.175
1,400 m	1.64 ± 0.22	1.62 ± 0.19			
Tubing assisted	1.59 ± 0.24	1.61 ± 0.19			
Paddle	1.60 ± 0.18	1.78±0.33			
Squat	1.39 ± 0.18	1.66±0.24			
Stroke frequency (Hz)			0.075	0.146	0.056
1,400 m	0.82 ± 0.11	0.81 ± 0.09			
Tubing assisted	0.80 ± 0.12	0.87 ± 0.16			
Paddle	0.82 ± 0.09	0.73 ± 0.13			
Squat	0.95 ± 0.12	0.81 ± 0.12			
25–50 m Stroke length (m)			<0.001*	0.015*	0.048*
1,400 m	1.61 ± 0.05	1.73 ± 0.19	1400 m > TA, *p* = 0.014	IM > BR	BR: SQ > 1400 m, *p* = 0.008**
Tubing assisted	1.47 ± 0.14	1.68 ± 0.17	PD > TA, *p* < 0.001**		BR: SQ > TA, *p* = 0.001**
Paddle	1.67 ± 0.10	1.78 ± 0.11	SQ > TA, *p* = 0.002**		BR: PD > TA, *p* = 0.001**
Squat	1.74 ± 0.13	1.74 ± 0.09			BR: 1400 m > TA, *p* = 0.012
					TA: IM > BR, *p* = 0.010**
Stroke frequency (Hz)			0.001*	0.055	0.005*
1,400 m	0.78 ± 0.05	0.74 ± 0.10	TA>1400 m, *p* = 0.010		BR: TA>1400 m, *p* = 0.005**
Tubing assisted	0.88 ± 0.07	0.75 ± 0.11	TA > PD, *p* = 0.009		BR: TA > PD, *p* = 0.014
Paddle	0.78 ± 0.06	0.73 ± 0.73	TA > SQ, *p* = 0.004**		BR: TA > SQ, *p* = 0.001**
Squat	0.72 ± 0.06	0.74 ± 0.05			BR: 1400 m > SQ, *p* = 0.004**
					BR: PD > SQ, *p* = 0.015
					TA: BR > IM, *p* = 0.005**
15–50 m Stroke length (m)			0.009*	0.005*	0.586
1,400 m	1.62 ± 0.09	1.69 ± 0.16	1400 m > TA, *p* = 0.035	IM > BR	
Tubing assisted	1.50 ± 0.15	1.66 ± 0.15	PD > TA, *p* = 0.005**		
Paddle	1.63 ± 0.10	1.77 ± 0.12	SQ > TA, *p* = 0.025		
Squat	1.62 ± 0.09	1.71 ± 0.08			
Stroke frequency (Hz)			0.016*	0.027*	0.294
1,400 m	0.79 ± 0.05	0.76 ± 0.09	TA>1400 m, *p* = 0.037	BR > IM	
Tubing assisted	0.86 ± 0.06	0.78 ± 0.10	TA > PD, *p* = 0.014		
Paddle	0.79 ± 0.05	0.73 ± 0.07	TA > SQ, *p* = 0.024		
Squat	0.78 ± 0.05	0.76 ± 0.05			

BR, Breaststroke; IM, Individual medley; TA, Tubing-assisted; PD, Paddle; SQ, Squat.

*p < 0.05, **p < 0.0083 (Bonferroni adjustment for post hoc comparison).

An analysis of segmental acceleration revealed that the data of the maximum forward acceleration of the hand did not exhibit a normal distribution. The Friedman test was used to analyze the differences among the four warm-up methods within each group of swimmers, and the Mann-Whitney U test was employed to compare the differences between the two groups across various warm-up methods. The results showed no significant differences in the maximum forward acceleration of the hand across the four warm-up methods within each group of swimmers. However, the comparison between breaststroke and medley specialists revealed that breaststroke specialists demonstrated faster maximum forward acceleration of the hand following TA warm-ups at the 15 m mark (*p* = 0.031), SQ warm-ups at the 25 m mark (*p* = 0.018), and after 1,400 m (*p* = 0.024) and SQ warm-ups (*p* = 0.009) near the 50 m destination. Additionally, at the 25 m mark, an interaction between the warm-ups and specialties for the maximum forward acceleration of the sacrum (F (3, 48) = 1.075, *p* = 0.038, *η*_*p*_^*2*^ = 0.067) and the maximum backward acceleration of the foot (F (3, 48) = 1.704, *p* = 0.032, *η*_*p*_^*2*^ = 0.557) was observed. Individual medley specialists exhibited higher maximum forward sacrum acceleration following the SQ warm-ups than following the TA warm-ups (*p* = 0.048). During the PD warm-ups, the maximum backward acceleration of the foot was significantly greater for breaststroke specialists than individual medley specialists (*p* = 0.009) (Table 4).

**TABLE 4:** Split acceleration of each body segment for 50 m breaststroke race.

Warm-ups	Breaststroke	Individual medley	Two-way ANOVA with mixed design (*p*-value)		
	(n = 9)	(n = 9)	Intervention	Group	Interaction
	Mean ± SD	Mean ± SD			Post hoc
Reach 15 m Max. forward acceleration of the hand (m/s^2^)					
1400 m	47.98 ± 15.23	34.03 ± 6.14			
Tubing assisted	48.88 ± 15.90	30.88 ± 8.37^a^			
Paddle	45.18 ± 15.26	33.95 ± 3.71			
Squat	45.78 ± 11.76	34.84 ± 7.59			
Max. backward acceleration of the hand (m/s^2^)			0.732	0.501	0.935
1400 m	25.84 ± 5.21	27.20 ± 2.53			
Tubing assisted	26.94 ± 9.09	29.41 ± 5.88			
Paddle	26.79 ± 4.26	27.36 ± 3.68			
Squat	26.49 ± 5.68	27.71 ± 2.59			
Max. forward acceleration of the sacrum (m/s^2^)			0.319	0.255	0.055
1,400 m	18.39 ± 4.47	22.06 ± 2.58			
Tubing assisted	19.62 ± 3.88	20.46 ± 3.07			
Paddle	20.28 ± 3.70	19.30 ± 3.43			
Squat	19.78 ± 4.24	23.06 ± 2.58			
Max. backward acceleration of the foot (m/s^2^)			0.115	0.057	0.109
1,400 m	62.51 ± 7.80	60.68 ± 11.39			
Tubing assisted	60.64 ± 9.04	54.01 ± 5.10			
Paddle	64.34 ± 6.12	52.30 ± 3.28			
Squat	63.98 ± 9.06	58.16 ± 9.02			
Reach 25 m Max. forward acceleration of the hand (m/s^2^)					
1,400 m	47.68 ± 17.27	34.72 ± 3.73			
Tubing assisted	46.28 ± 18.67	34.38 ± 6.56			
Paddle	47.68 ± 17.27	34.72 ± 3.73			
Squat	44.13 ± 12.19	35.21 ± 3.66^a^			
Max. backward acceleration of the hand (m/s^2^)					
1,400 m	25.17 ± 4.55	28.89 ± 3.70	0.287	0.112	0.559
Tubing assisted	26.00 ± 8.63	30.17 ± 4.64			
Paddle	26.51 ± 2.28	28.89 ± 3.70			
Squat	26.29 ± 5.18	29.71 ± 4.28			
Max. forward acceleration of the sacrum (m/s^2^)					
1,400 m	19.52 ± 5.95	24.34 ± 3.45	0.395	0.309	0.038*
Tubing assisted	22.44 ± 6.77	21.33 ± 3.73			IM: SQ > TA, *p* = 0.048
Paddle	20.67 ± 4.71	20.30 ± 3.12			
Squat	20.33 ± 5.37	25.09 ± 4.82			
Max. backward acceleration of the foot (m/s^2^)					
1,400 m	61.53 ± 6.57	57.75 ± 16.94	0.369	0.194	0.032*
Tubing assisted	63.75 ± 10.84	60.18 ± 10.74			PD: BR > IM, *p* = 0.009
Paddle	65.51 ± 5.13	53.32 ± 11.47			
Squat	64.38 ± 7.98	60.08 ± 11.22			
Reach 50 m Max. forward acceleration of the hand (m/s^2^)					
1,400 m	44.23 ± 15.66	29.14 ± 4.67^a^			
Tubing assisted	41.21 ± 16.04	27.64 ± 7.89			
Paddle	40.78 ± 17.15	31.31 ± 3.28			
Squat	42.97 ± 12.32	29.13 ± 4.36^a^			
Max. backward acceleration of the hand (m/s^2^)					
1,400 m	24.31 ± 5.02	25.99 ± 4.30	0.687	0.730	0.413
Tubing assisted	24.44 ± 6.45	26.19 ± 2.50			
Paddle	24.03 ± 6.62	24.06 ± 5.30			
Squat	24.54 ± 5.53	24.27 ± 4.63			
Max. forward acceleration of the sacrum (m/s^2^)					
1,400 m	17.24 ± 4.63	21.30 ± 3.41	0.892	0.409	0.093
Tubing assisted	18.56 ± 4.74	18.63 ± 2.34			
Paddle	18.91 ± 4.13	18.71 ± 1.08			
Squat	18.04 ± 4.89	20.14 ± 3.38			
Max. backward acceleration of the foot (m/s^2^)					
1,400 m	61.65 ± 5.44	60.95 ± 6.57	0.390	0.556	0.331
Tubing assisted	56.18 ± 9.19	56.50 ± 10.12			
Paddle	60.99 ± 6.62	56.71 ± 5.11			
Squat	62.13 ± 8.28	59.38 ± 5.47			

BR, Breaststroke; IM, Individual medley; TA, Tubing assisted; PD, Paddle; SQ, squat.

*p < 0.05, **p < 0.0083 (Bonferroni adjustment for *post hoc* comparison).

The data of the Max. forward acceleration of the hand did not exhibit a normal distribution. Non-parametric analysis was employed.

^a^ Indicates significant differences between breaststroke and medley specialists as determined by the Mann-Whitney U test.

## 4 Discussion

The various warm-ups induced varying effects on breaststroke and individual medley specialists, particularly in changes in stroke length and stroke frequency beyond the 25 m mark. The test results indicate that the TA warm-ups significantly improved stroke frequency, enhancing the 50 m breaststroke performance of breaststroke specialists. Resistance-based PD warm-ups also modestly improved their performance. These findings are consistent with those of studies in which warm-ups mimicked swimming motions (Hancock et al., 2015; McGowan et al., 2016). Both dryland strength warm-ups simulating the swimming kinetic chain and using a resistive power rack to provide resistance in water for repeated sprint warm-ups showed significantly better performance in the 100 m freestyle. However, other studies have demonstrated no significant improvement in swimming performance following specialized warm-ups such as 5 repetitions of the inertial flywheel or Smith machine or using one set of three reps at 87% 1RM back squats as a warm-up (Cuenca-Fernández et al., 2020; Kilduff et al., 2011; Sarramian et al., 2015). This discrepancy may be due to these studies requiring participants to engage in resistance-based warm-ups to recruit muscle fibers to improve performance. Sharma et al. (2018) noted that resistance-based warm-ups may cause inconsistent fatigue dissipation durations compared with bodyweight plyometric warm-ups. Consequently, although resistance-based warm-ups can enhance strength and power, they may also result in residual fatigue that outweighs performance benefits. Therefore, when implementing resistance-based warm-ups, it is essential to consider the athletes’ individual capabilities to avoid performance being negatively impacted by slower fatigue recovery. In this study, whether providing a similar amount of resistance-based warm-ups to each athlete resulted in varying levels of fatigue, thereby diminishing the effectiveness of such warm-ups, particularly in the case of SQ warm-ups, remains unclear and warrants further investigation. Studies have verified that assisted warm-ups can enhance both jumping and sprinting performance on land, and such warm-ups are also widely performed in competitive swimming. However, no studies have directly verified the benefits of assisted warm-ups on swimming performance (Cazas et al., 2013; Nealer et al., 2017). Unlike other warm-ups, the TA warm-ups reduce resistance and enable swimmers to achieve an overspeed effect (Sheppard et al., 2011; Stien et al., 2020). This can result in faster recruitment of motor units and increase movement speed and frequency (Cazas et al., 2013). This means that it enhances the speed-strength portion of the force-velocity curve, emphasizing the speed of recruitment over the recruitment of more motor units (Cross et al., 2017). An overspeed effect was also observed in the present study, in which the stroke frequency of swimmers performing TA warm-ups from 15 to 50 m was significantly higher than that of those performing the other three warm-up methods. This effect was particularly evident in breaststroke specialists, who exhibited a significantly higher stroke frequency and shorter time in the final 25 m after engaging in TA warm-ups compared with the conventional 1,400 m warm-up. This result indicates that the TA warm-ups increased stroke frequency and synergized with breaststroke specialists’ strategy of using a high stroke frequency during the 50 m breaststroke sprint. Additionally, in breaststroke specialists, the SQ and PD warm-ups resulted in longer stroke lengths in the final 25 m, whereas the TA warm-ups resulted in the shortest stroke length. This discrepancy may be due to the resistance-based nature of both the SQ and PD warm-ups, which can improve the neuromuscular recruitment of motor units, enabling swimmers to gain strength after a brief rest (Tillin and Bishop, 2009) and resulting in increased distance covered per stroke. This approach leans more towards the strength-speed portion of the force-velocity curve, focusing on recruiting more motor units to generate power rather than speed. As a result, each stroke covers a longer distance, but it also comes at the cost of a reduced stroke frequency (Cross et al., 2017).

The two resistance-based warm-up methods in this study had varying objectives. Specifically, the SQ warm-up, performed at 85% of 1 RM for five repetitions per set, focused on developing lower limb strength rather than frequency to increase the efficiency of each kick. By contrast, the PD warm-up targeted the upper limbs, utilizing a posture closer to the actual breaststroke to enhance upper limb strength and improve the propulsion of each stroke. The results indicate that both resistance-based warm-ups resulted in a slight increase in stroke length particularly between 25 and 50 m, but a decrease in stroke frequency was also observed, leading to no significant change in time during this interval for both groups of swimmers.

Breaststroke specialists engage in specific and intensive training for the breaststroke, leading to increased mastery, strength control, frequency, and proficiency in this stroke compared with individual medley swimmers. By contrast, individual medley swimmers, who primarily compete in 200 m events, often adopt a gliding strategy to conserve energy during the 50 m breaststroke. This difference results in variations in frequency and movement control between the groups. Thus, breaststroke and individual medley swimmers should employ different warm-ups. For breaststroke specialists, who prefer a high frequency strategy for the 50 m breaststroke, the TA warm-ups, which enhance movement speed and frequency, most significantly improved performance. By contrast, for individual medley swimmers, whose stroke efficiency and kick propulsion require improvement, the SQ warm-up led to slight improvements. Further studies are required to determine whether varying resistance loads and repetitions yield improved results for individual medley swimmers.

Other studies have verified the accuracy of IMUs in measuring instantaneous speed and intracycle variability in swimmers. However, no studies have compared differences in the center of mass and limb segment accelerations between swimmers of varying specialties employing varied interventions and segments using IMUs (Dadashi et al., 2012; Hamidi Rad et al., 2022). Studies have demonstrated that propulsion in breaststroke primarily results from the upper limb pull during the upper limb propulsion phase and the whip kick during the lower limb propulsion phase. Additionally, reducing the time required for the upper limb recovery phase, in which the hand returns to a position parallel to the head with the elbow fully extended, can accelerate the swimming cycle (Strzala et al., 2013; Strzała et al., 2012). To investigate these variations more accurately and conveniently, this study attached IMUs to the hands, sacrum, and feet of the swimmers to measure acceleration during the propulsion and recovery phases. Analyzing movements at approximately 15, 25, and 50 m, We observed that breaststroke specialists exhibited significantly higher maximum forward hand accelerations under certain warm-up conditions during the 50 m breaststroke compared to individual medley specialists. This finding is consistent with our previous observations and suggests that breaststroke specialists prefer shorter stroke cycles to increase frequency during the 50 m breaststroke. At the 25 m mark, the IMU data revealed that the individual medley specialists had a faster forward sacrum acceleration following the SQ warm-up, indicating that this warm-up improved their propulsion. Additionally, following the PD warm-up, the breaststroke specialists had a higher maximum backward foot acceleration than the individual medley specialists, indicating superior lower limb propulsion by the breaststroke specialists. However, these findings were insufficient to fully explain the variations in stroke frequency, stroke length, and overall performance resulting from the various warm-up protocols. Future studies should include an analysis of the movement angles and angular accelerations of each limb segment and incorporate electromyographic analysis to provide additional insights into the neuromuscular adaptations that breaststroke specialists undergo during the TA warm-ups, which increase stroke frequency and enhance performance in the 50 m breaststroke.

By observing the stroke length and frequency of breaststroke and individual medley swimmers during the 50-m breaststroke race, we could identify the factors influencing swimming performance, as a previous study mentioned (Simbaña-Escobar et al., 2020). The association between stroke length and frequency is analogous to that of stride length and frequency in running; although an inverse relationship exists between these two variables, increasing either can enhance performance (Gonjo et al., 2022; Hunter et al., 2004). Additionally, one study comparing 200 m breaststroke specialists with individual medley swimmers demonstrated that, due to energy expenditure over the longer distance, breaststroke specialists have a longer stroke length without a significant difference in frequency. This finding indicates that breaststroke specialists exhibit superior propulsion efficiency (Gonjo et al., 2022). By contrast, the experiment of the present study focused on the 50 m breaststroke, a distance for which energy expenditure is less critical. Throughout the experiment, breaststroke specialists generally emphasized increasing stroke frequency to achieve acceleration, a common strategy for enhancing speed (Barbosa et al., 2008; Takagi et al., 2023). However, individual medley swimmers tended to adopt a larger stroke length to compensate for their insufficient thrust and low propulsion efficiency.

This study has several limitations. First, the small sample size and unequal sex distribution across groups reduce statistical power, limit generalizability, and require cautious interpretation. Second, unlike official competitions where athletes compete against each other, this experiment used individual timed trials, which may have influenced the swimmers’ psychological preparedness. Third, attaching IMUs to the swimmers may have affected water resistance and their perception of the swimming experience.

## 5 Conclusion

This study revealed differences in the 50 m breaststroke strategies of breaststroke specialists and individual medley specialists. Specifically, breaststroke specialists exhibited a higher stroke frequency, whereas individual medley specialists had a longer stroke length. Although both groups achieved similar results in the 50 m breaststroke, the effects of the various warm-ups varied. Breaststroke specialists exhibited significant improvement in their 50 m breaststroke performance after the TA warm-up. By contrast, individual medley specialists benefited more from the SQ warm-up. The improvement observed in breaststroke specialists was attributed to the increased stroke frequency induced by the TA warm-up, whereas the squat warm-up enhanced lower limb power in the individual medley specialists, improving an area of weakness unique to them. The use of IMUs to measure limb segment accelerations and center of mass acceleration provided insights into the variations in propulsion and recovery movements and forward acceleration during swimming. These data enable rapid analysis and presentation compared with conventional 2D video analysis, substantially reducing the time required to obtain and process data.

## Data Availability

The original contributions presented in the study are included in the article/supplementary material, further inquiries can be directed to the corresponding author.
